# Efficacy, immunogenicity, and safety of IC43 recombinant *Pseudomonas aeruginosa* vaccine in mechanically ventilated intensive care patients—a randomized clinical trial

**DOI:** 10.1186/s13054-020-2792-z

**Published:** 2020-03-04

**Authors:** Christopher Adlbrecht, Raphael Wurm, Pieter Depuydt, Herbert Spapen, Jose A. Lorente, Thomas Staudinger, Jacques Creteur, Christian Zauner, Andreas Meier-Hellmann, Philipp Eller, Margot Vander Laenen, Zsolt Molnár, István Várkonyi, Bernhard Schaaf, Mária Héjja, Vladimír Šrámek, Hauke Schneider, Niranjan Kanesa-thasan, Susanne Eder-Lingelbach, Anton Klingler, Katrin Dubischar, Nina Wressnigg, Jordi Rello

**Affiliations:** 1 Department of Cardiology, Vienna North Hospital–Clinic Floridsdorf and the Karl Landsteiner Institute for Cardiovascular and Critical Care Research, Vienna, Austria; 2 Medical University of Vienna, Vienna, Austria; 3 Universitair Ziekenhuis Gent, Ghent, Belgium; 4 Universitair Ziekenhuis Brussel, Brussels, Belgium; 5 Hospital Universitario de Getafe, CIBER de Enfermedades Respiratorias, Universidad Europea, Madrid, Spain; 6 Erasme University Hospital, Brussels, Belgium; 7 HELIOS Klinikum Erfurt GmbH, Erfurt, Germany; 8 Medical University of Graz, Graz, Austria; 9 Ziekenhuis Oost-Limburg, Campus St. Jan, Genk, Belgium; 10 Szegedi Tudományegyetem, Szeged, Hungary; 11 Kenézy Kórház Debrecen, Debrecen, Hungary; 12 Klinikum Dortmund GmbH, Dortmund, Germany; 13 Országos Korányi TBC és Pulmonológiai Intézet, Budapest, Hungary; 14 Fakultní nemocnice U Svaté Anny v Brně, Brno, Czech Republic; 15 Technische Universität Dresden, Dresden, Germany; 16 University Hospital Augsburg, Augsburg, Germany; 17 GSK Vaccines, Rockville, MD USA; 18 Valneva Austria GmbH, Campus Vienna Biocenter 3, 1030 Vienna, Austria; 19 Assign Data Management and Biostatistics GmbH, Innsbruck, Austria; 20 Centro de Investigacion Biomedica en Red (CIBERES), Hospital Universitari Vall d’Hebron, Barcelona, Spain

**Keywords:** *Pseudomonas aeruginosa*, Vaccination, Intensive care, Mechanical ventilation

## Abstract

**Background:**

*Pseudomonas aeruginosa* infections are a serious threat in intensive care units (ICUs). The aim of this confirmatory, randomized, multicenter, placebo-controlled, double-blind, phase 2/3 study was to assess the efficacy, immunogenicity, and safety of IC43 recombinant *Pseudomonas aeruginosa* vaccine in non-surgical ICU patients.

**Methods:**

Eight hundred patients aged 18 to 80 years admitted to the ICU with expected need for mechanical ventilation for ≥ 48 h were randomized 1:1 to either IC43 100 μg or saline placebo, given in two vaccinations 7 days apart. The primary efficacy endpoint was all-cause mortality in patients 28 days after the first vaccination. Immunogenicity and safety were also evaluated.

**Findings:**

All-cause mortality rates at day 28 were 29.2% vs 27.7% in the IC43 and placebo groups, respectively (*P* = .67). Overall survival (Kaplan-Meier survival estimates, *P* = .46) and proportion of patients with ≥ one confirmed *P. aeruginosa* invasive infection or respiratory tract infection also did not differ significantly between both groups. The geometric mean fold increase in OprF/I titers was 1.5 after the first vaccination, 20 at day 28, after the second vaccination, and 2.9 at day 180. Significantly more patients in the placebo group (96.5%) had ≥ one adverse event (AE) versus the IC43 100 μg group (93.1%) (*P* = .04). The most frequently reported severe AEs in the IC43 and placebo groups were respiratory failure (6.9% vs 5.7%, respectively), septic shock (4.1% vs 6.5%), cardiac arrest (4.3% vs 5.7%), multiorgan failure (4.6% vs 5.5%), and sepsis (4.6% vs 4.2%). No related serious AEs were reported in the IC43 group.

**Interpretation:**

The IC43 100 μg vaccine was well tolerated in this large population of medically ill, mechanically ventilated patients. The vaccine achieved high immunogenicity but provided no clinical benefit over placebo in terms of overall mortality.

**Trial registration:**

https://clinicaltrials.gov (NCT01563263). Registration was sent to ClinicalTrials.gov on March 14, 2012, but posted by ClinicalTrials.gov on March 26, 2012. The first subject was included in the trial on March 22, 2012.

## Introduction

Nosocomial infections are an increasing problem in intensive care units (ICUs), with infection rates reportedly two to five times higher than in the general inpatient hospital population [1]. Elderly patients who are multimorbid, immunocompromised, and increasingly vulnerable to antibiotic-resistant bacteria are particularly at risk [2].

*Pseudomonas aeruginosa* is an invasive, gram-negative bacterium that is known to cause a wide range of acute and chronic infections [3]. *P. aeruginosa* rarely causes infection in the healthy host, yet it is an efficient opportunistic pathogen that can cause serious infections in patients who have weakened immune systems, e.g., individuals who are immunocompromised, and patients with malignancies or HIV infection [4]. *P. aeruginosa* is one of the most common nosocomial pathogens and is a major cause of morbidity and mortality for hospitalized patients [5]. It is responsible for approximately 10% of all hospital-acquired infections and has an estimated mortality rate of up to 50% [5, 6]. It is common in ICU patients with chronic disease and/or those who acquire ventilator-associated pneumonia [7–10]. Despite the availability of new classes of antibiotics, multidrug resistance to *P. aeruginosa* has doubled over the past 30 years [6–11]. Thus, new approaches are required to prevent and cure *P. aeruginosa* infections. Vaccination strategies that may confer long-lived adaptive immunity against multidrug-resistant, gram-negative bacterial infections have produced mixed results [12]. However, the investigational vaccine IC43 appeared to be promising based on the favorable safety and immunogenicity profile from previous phase 1/2 studies [13, 14]. In the phase 2 study, IC43 demonstrated a significant immunogenic effect in ventilated ICU patients without safety concerns. In addition, all-cause mortality was reduced versus placebo (more pronounced in patients with infections), although there were no significant differences in *P. aeruginosa* infection rates [13].

In the current study, we sought to confirm the previously observed effects of IC43 100 μg on all-cause mortality in a large, high-risk ICU population of medically ill, intubated patients with the need for mechanical ventilation (MV).

## Methods

### Study design

This was a confirmatory, randomized, multicenter, placebo-controlled, double-blind phase 2/3 study to assess the efficacy, immunogenicity, and safety of IC43 recombinant *P. aeruginosa* vaccine in ICU patients, with a follow-up duration of 180 days after the first vaccination (NCT01563263).

The study was conducted from 2012 to 2015 at 52 study sites in six European countries (Austria, Belgium, Czech Republic, Germany, Hungary, and Spain). The study was approved by the responsible Ethics Committees and compliant with the current International Conference on Harmonization/Good Clinical Practice guidelines and in accordance with the principles set forth in the Declaration of Helsinki.

### Patients

Medically ill patients admitted to an ICU with the expected need for MV for ≥ 48 h who were aged 18 to 80 years at screening were eligible for inclusion. Female patients were only included if they lacked childbearing potential or had a negative pregnancy test. Written informed consent was obtained in line with local regulations. Exclusion criteria included a Sequential Organ Failure Assessment (SOFA) score < 4 at day 0 (day of screening), a previous organ transplant within the past 6 months, readmission to the ICU during the current total hospital stay, admission to the ICU within 2 days after surgery, admission to the ICU due to trauma, elective surgery until day 28 after the first vaccination, and expected plasmapheresis or immunoadsorption. The use of antibiotics was left to the discretion of the local physicians treating the patients.

### Interventions

Patients were randomized 1:1 to either receive IC43 100 μg (recombinant *P. aeruginosa* fusion protein construct consisting of epitopes of OprF and OprI with an N-terminal His 6 tag: Met-Ala-(His)6 – OprF 190–342 – OprI 21–83 protein [Valneva Austria GmbH, Vienna, Austria]) [15] or placebo (phosphate-buffered saline solution containing 0.9% NaCl). Each patient was planned to receive a total of two intramuscular vaccinations (of 1 mL in the deltoid muscle of the same upper arm) 7 days apart, on days 0 and 7.

### Study outcomes

The primary efficacy endpoint was all-cause mortality at day 28 in patients receiving either IC43 100 μg or placebo. Secondary efficacy endpoints included all-cause mortality at days 14, 56, and 90; all-cause mortality at days 28, 56, and day 90 in patients surviving through day 14; all-cause mortality at days 14, 28, 56, and 90 in patients surviving through day 3; overall survival; and the proportion of patients who had a *P. aeruginosa* infection/colonization within 28 and 56, 90, and 180 days after first vaccination; further pre-planned statistical analyses provided infection rates between day 7 and day 14 and day 14 and day 90.

*P. aeruginosa* infections were diagnosed by the investigator according to predefined diagnostic criteria [8, 16] and confirmed by an independent data monitoring committee (DMC). For the collection of *P. aeruginosa* infection data, investigators obtained cultures from blood samples, central venous catheter device samples, respiratory secretions, and urine as medically indicated, according to the local standard of care. The fact that there was no convincing signal concerning an effect of the vaccine evaluated on *P. aeruginosa* infections in the phase 2 trial in addition to the difficulties in interpreting over-infection led to the decision not to screen per protocol for infections in the phase 3 trial. Investigators documented *P. aeruginosa* infections (e.g., bacteremia, urinary tract infection, pneumonia, tracheobronchitis) or respiratory tract colonization. Organ function (SOFA scores) was assessed at all visits performed at the ICU. In addition, the length of ICU stay after the first vaccination and overall hospital stay were evaluated. Immunogenicity at days 7, 14, 28, 56, and 180 was determined by measuring OprF/I-specific immunoglobulin G antibody titer using an enzyme-linked immunosorbent assay. Safety was evaluated up to 180 days after the first vaccination, including adverse events (AEs), serious AEs, and laboratory parameters; systemic and local tolerability were assessed during up to 7 days post vaccination.

### Statistical analysis

A sample size of 800 patients (400 per group) was selected to allow for the detection of a 10% difference in mortality rates with a power ≥ 90% and an expected mortality rate in the placebo group of 25 to 30% (two-sided significance level of 5%; *χ*^2^ test without continuity correction). Patients were randomized 1:1 into one of the treatment groups using an ascending Investigational Medicinal Product (IMP) kit number, where IMP kits were prepacked according to the randomization plan generated before the study starts with a block size of four (two per group). The primary efficacy analysis compared the difference in day 28 all-cause mortality (number of deaths until day 28/total number of observed patients in treatment group) between the two treatment groups in the intent-to-treat analysis population (ITT, all randomized patients), using a Cochran-Mantel-Haenszel test stratified for the country with the null hypothesis of no difference between treatment groups and a significance level of 5% (two-sided). In addition, logistic regression was applied to assess the independent influence of treatment with covariates such as the country, patient’s age, SOFA score, and the Acute Physiology and Chronic Health Evaluation (APACHE) II score as covariates. Survival endpoints were evaluated using Kaplan-Meier curves and Cox regressions, with the factors treatment group, country, patient’s age, and SOFA/APACHE II score at day 0. SOFA scores after day 0 (visit 0) were compared between treatment groups for each visit using a van Elteren test stratified for the country. Geometric mean titers and geometric mean titer ratios were estimated by applying an analysis of variance including the factors country and vaccination group after log_10_ transformation. The geometric mean fold change of OprF/I titers was also estimated. All patients who received at least one vaccination and provided postvaccination safety data were included in the safety analysis.

After enrollment of 50% of patients, i.e., after 400 patients, an interim analysis for futility was planned to compare the day 28 mortality between the IC43 100 μg group and placebo group and to allow discontinuation of the study in case of insufficient vaccine effect. A conditional power at the end of the trial of at least 60% was used as the nonbinding futility criterion. All the study staff including statisticians remained blinded for treatment allocation during the interim analysis, which was reviewed exclusively by the DMC and the study steering committee.

SAS 9.3 TS1M2 (second maintenance release) was used.

## Results

**Fig. 1:**
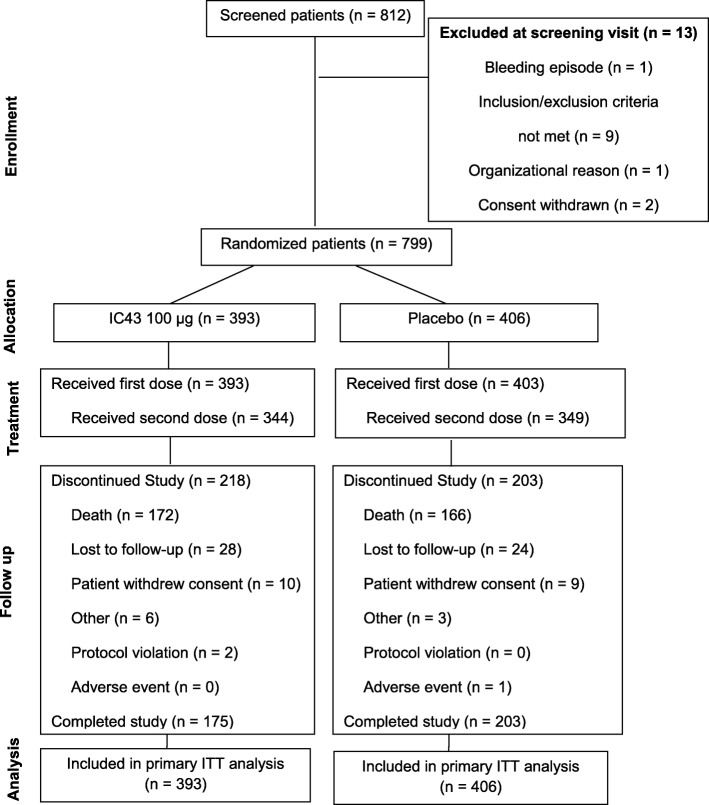
CONSORT diagram

Between 2012 and 2015, a total of 812 patients were enrolled at 52 study centers (Fig. 1); 799 patients were randomized to receive either IC43 100 μg (*N* = 393) or placebo (*N* = 406). The ITT population included all randomized patients. The number of patients who received the second injection (IMP or PBS) was 344 (87.5%) in the IC43 and 349 (86.0%) in the placebo group. Seven hundred ninety-six patients who received at least one IMP injection were included in the safety analysis.

The baseline characteristics of the study population are presented in Table 1. There were no major differences in terms of medical history between the two treatment groups. The most common concomitant medications were antibiotics for systemic use (98.2% [*n* = 782] and antithrombotic agents (90.7% [*n* = 722]). Among the 629 patients (79.0%) treated with *Pseudomonas*-specific anti-infective therapy, carbapenems were the most frequently reported (41.3% [*n* = 329]). The number of days on antibiotic treatment was similar in the IC43 100 μg and placebo groups (mean 54.4 vs 52.0 days; median 16 days in both groups). The use of systemic antibiotic treatments before and after the second IMP injection was similar in the IC43 and placebo groups when pooled across all countries (85.2% vs 83.6% and 63.4% vs 59.6%, respectively). The number of patients receiving *P. aeruginosa* relevant antibiotics at baseline or prior to inclusion was very high (79%), being consistent with the literature [10, 17]. No additional post hoc analysis of the group without *P. aeruginosa* active antibiotics has been performed due to a small sample size. The following antibiotics were considered relevant for *P. aeruginosa*: piperacillin, piperacillin/tazobactam, ceftazidime, ceftirome, cefepime, group 1 carbapenems (imipenem, meropenem, doripenem), aminoglycosides, ciprofloxacin, levofloxacin, and aztreonam.

**Table 1:** Baseline characteristics per treatment group (safety population)

	IC43 100 μg (*n* = 393)No. (%)	Placebo (*n* = 403)No. (%)	Total (*N* = 796)No. (%)
Age, mean, years (min/max)	61.8 (18/86^a^)	62.0 (18/80)	61.9 (18/86)
Sex, no. (%)			
Male	264 (67.2)	269 (66.7)	533 (67.0)
Female	129 (32.8)	134 (33.3)	263 (33.0)
BMI	28.9	28.5	28.7
Ethnicity, no. (%)			
Caucasian	387 (98.5)	395 (98.0)	782 (98.2)
Asian	1 (0.3)	1 (0.2)	2 (0.3)
Black	4 (1.0)	5 (1.2)	9 (1.1)
Other	1 (0.3)	2 (0.5)	3 (0.4)
SOFA score, mean (±SD)	8.1 (± 2.9)	8.3 (± 2.8)	8.2 (± 2.8)
APACHE II score, mean (±SD)	18.7 (± 7.2)	19.8 (± 7.9)	19.2 (± 7.6)
Medical history (reported events > 15%), no. (%)			
Hypertension	220 (56.0)	225 (55.8)	445 (55.9)
Pneumonia	160 (40.7)	162 (40.2)	322 (40.5)
Chronic obstructive pulmonary disease	92 (23.4)	105 (26.1)	197 (24.7)
Respiratory failure	78 (19.8)	91 (22.6)	169 (21.2)
Acute kidney injury	76 (19.3)	87 (21.6)	163 (20.5)
Atrial fibrillation	72 (18.3)	82 (20.3)	154 (19.3)
Obesity	71 (18.1)	69 (17.1)	140 (17.6)
Sepsis	70 (17.8)	52 (12.9)	122 (15.3)
Diabetes mellitus	68 (17.3)	66 (16.4)	134 (16.8)
Septic shock	63 (16.0)	72 (17.9)	135 (17.0)
Anemia	59 (15.0)	72 (17.9)	131 (16.5)
Hypotension	57 (14.5)	69 (17.1)	126 (15.8)
Prior medication^b^			
Antibacterials for systemic use	177 (45.0)	184 (45.7)	361 (45.4)
Antidiarrheals, intestinal anti-inflammatories	16 (4.1)	12 (3.0)	28 (3.5)
Antimycobacterials	7 (1.8)	8 (2.0)	15 (1.9)

*Abbreviations*: *BMI* body mass index, *ICU* intensive care unit, *Q* quarter, *SOFA* Sequential Organ Failure Assessment

^a^Five patients aged > 80 years were vaccinated; these constituted a major protocol deviation and were subsequently omitted from the per-protocol analysis

^b^Patients are counted only once per Anatomical Therapeutic Chemical level 2 (World Health Organization Drug Dictionary Q4_2015).

^c^Intent-to-treat population (IC43: *n* = 393; placebo: *n* = 406)

### Primary efficacy endpoint

**Fig. 2:**
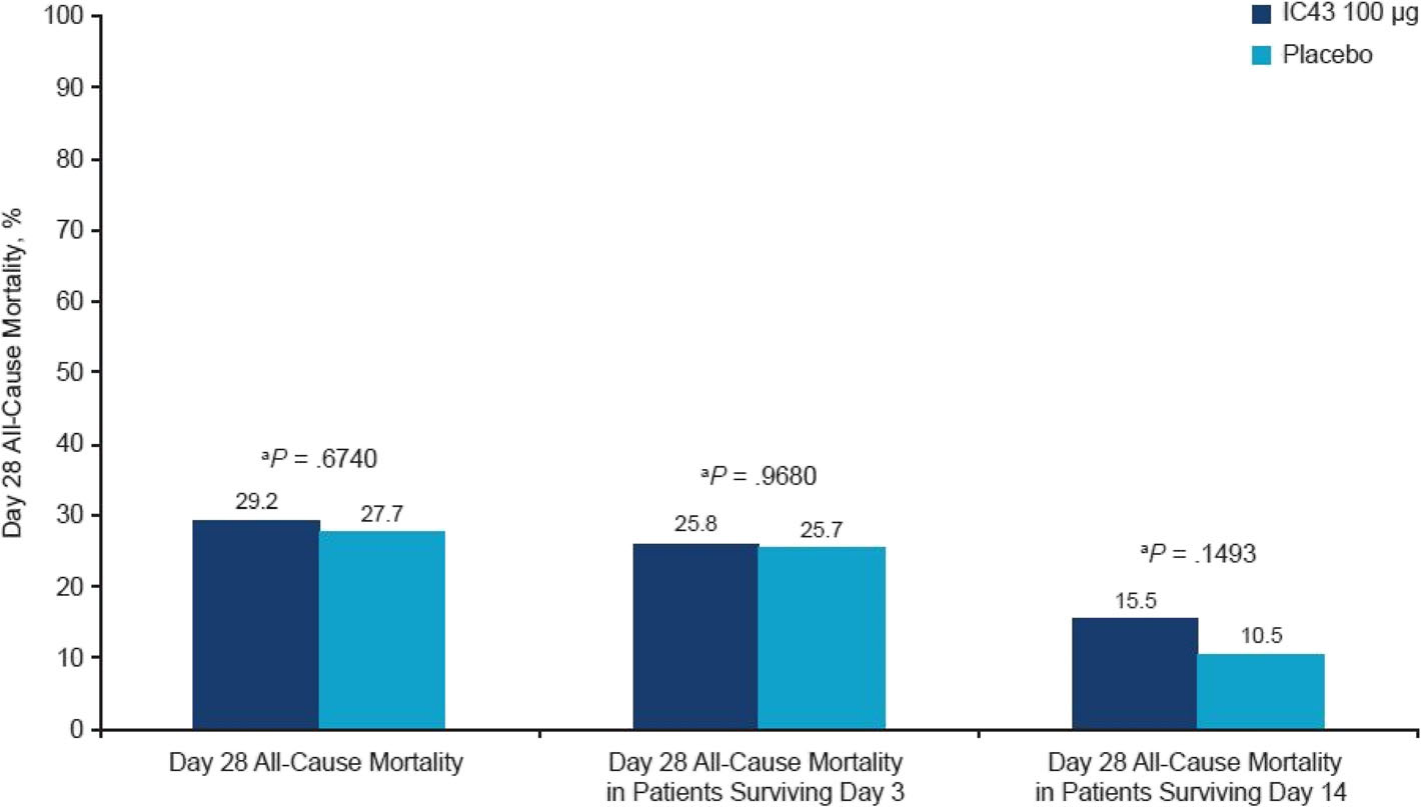
All-cause mortality (intent-to-treat population). ^a^IC43 100 μg versus placebo

Overall, all-cause mortality rates were similar in the two treatment arms; the proportion of patients who died through day 28 was 29.2% (108/370 [95% confidence interval (CI), 24.8–34.0]) in the IC43 100 μg group and 27.7% (108/390 [95% CI, 23.5–32.3]) in the placebo groups (*P* = .6740) (Fig. 2). However, there were substantial differences in all-cause mortality rates at day 28 between the different countries (Austria:29.5%, Belgium 33.8%, Hungary 35.0%, Germany 24.1%, Spain 18.5%, and Czech Republic 14.0%, for the total study population).

For the interim analysis (*N* = 394), the pre-specified, nonbinding futility criterion was formally met; however, the reduction in day 28 all-cause mortality between the IC43 100 μg (28.3%) and placebo (32.8%) groups was considered clinically meaningful by the DMC, and hence, the study was continued.

### Secondary efficacy endpoints

In patients surviving through day 3, all-cause mortality rates were similar in the IC43 and placebo groups (Fig. 2); however, in patients who survived through day 14, all-cause mortality rates were higher in the IC43 group vs the placebo group, but the differences were not significant (Fig. 2).

**Fig. 3:**
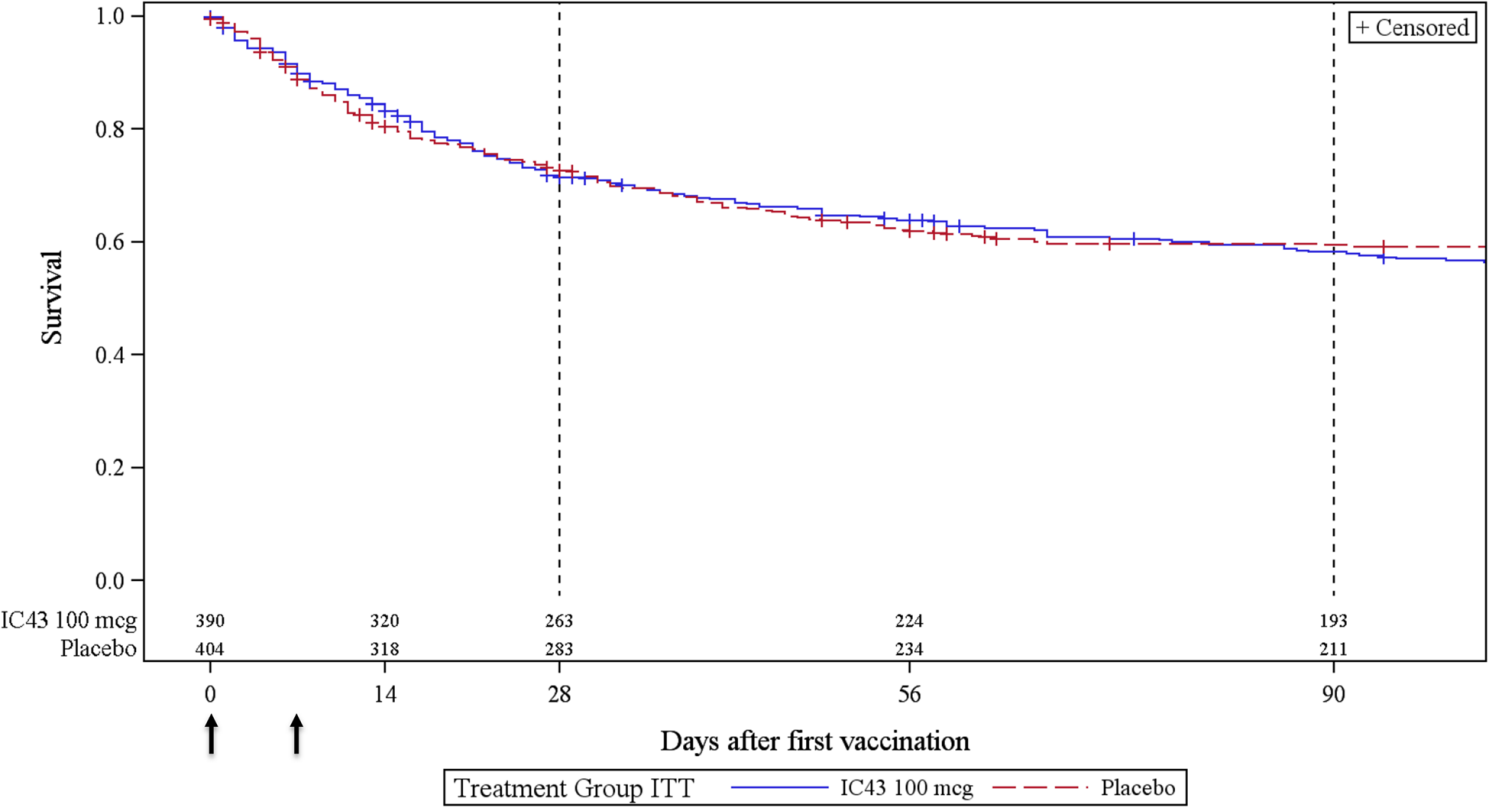
Overall survival: Kaplan-Meier curves by treatment group (intent-to-treat population). Arrows depict vaccination time points. Numbers reflect a number of subjects at risk. + Symbols denote censored patients

Overall survival (Kaplan-Meier survival estimates) did not differ significantly between the groups (*P* = .4603) (Fig. 3). Mortality rates were numerically slightly lower in the IC43 group than in the placebo group on day 14 (17.1 vs 19.8%) and day 56 (37.6 vs 38.9%) and higher on day 90 (44.4 vs 42.7%) and day 180 (60.6 vs 55.3%); again, these differences were not significant.

Based on the Cox regression models, there was no significant impact of treatment group on survival; however, baseline SOFA score alone was negatively associated with survival, independent of treatment group (*P* < .0001). Concomitant corticosteroid use also significantly affected survival, regardless of treatment group (*P* < .05) or treatment group plus SOFA score (*P* < .05).

At all visits between days 0 and 180, mean SOFA scores were similar in the IC43 100 μg (range, 2.0–8.1) and placebo (range, 3.4–8.2) groups (eTable 2 in the Supplement). The mean length of ICU stay after the first IMP injection was also similar between treatments (mean 22.3 vs 22.4 days; IQR, 19.0 vs 19.5 days), as was the length of hospital stay (33.6 vs 34.5 days; IQR, 32 vs 30 days).

The rates of patients with at least one DMC-confirmed *P. aeruginosa* invasive infection or respiratory tract infection (e.g., pneumonia, tracheobronchitis) starting between days 14 and 90 did not differ significantly between the IC43 100 μg and placebo groups (eTable 1 in the Supplement). In addition, no statistically significant differences were seen for the other periods (e.g., day 0 to day 7). Overall, the rates of *P. aeruginosa* events were not significantly different during the course of the study between the vaccination group and the placebo group respectively (bacteriemia 8 (2.0)% versus 11 (2.7%), *P* = 0.644; pneumonia 22 (5.6%) versus 32 (7.9%), *P* = 0.208; and urinary tract infection 7 (1.8%) versus 8 (2%) *P* = 1.000). *P. aeruginosa* respiratory infections developed a median 10.8 and 11 days after IMP injection in the IC43 100 μg and placebo groups, respectively. Rates of patients with at least one DMC-confirmed *P aeruginosa* respiratory tract colonization starting between days 0 and 7, between days 0 and 28, and between day 0 and study’s end differed significantly between the IC43 100 μg and placebo groups (5.1% vs 2.0%, *P* = .0201; 8.4% vs 4.7%, *P* = .0438; 10.7% vs 6.4%, *P* = .0316, respectively). No significant differences in DMC-confirmed *P. aeruginosa* respiratory tract colonization were observed between days 14 and 90 (*P* = .4591). Any *P. aeruginosa* respiratory tract colonization was detected 48 (12.2%) of the vaccination group and in 30 (7.4%) of the placebo group (*P* = 0.024). This difference likely represents a play of chance. Specific information on susceptibility to antibiotics was not prospectively collected in this trial and is therefore unfortunately not available to answer this question.

There was a clear difference with > 70% of patients immunized with IC43 100 μg attaining peak OprF/I-specific antibody titers > 1000 endotoxin units per milliliter at day 28. The findings from the phase 2 study were confirmed with the geometric mean fold increase in OprF/I titers ranging from 1.5 after the first vaccination to a peak of 20 on day 28 after the second vaccination and 2.9 at day 180. A detailed summary of the immunogenicity findings is included in Additional file 1.

A total of 5510 AEs were reported for 755 patients (Table 2). The number of patients with at least one AE was significantly higher in the placebo group (96.5%) than in the IC43 100 μg group (93.1%) (*P* = .0365). The most frequently reported AE (> 10% in any treatment group) was diarrhea (109 patients; 13.7%) (Table 2).

**Table 2:** Incidence of adverse events per treatment arm

	IC43 100 μg (*n* = 393)No. (%)	Placebo (*n* = 403)No. (%)	*P* value	Total (*N* = 796)No. (%)
Patients with ≥ 1 AE	366 (93.1)	389 (96.5)	.0365	755 (94.8)
Most frequently reported AEs by preferred term (> 10%)^a^				
Diarrhea	57 (14.5)	52 (12.9)	.5371	109 (13.7)
Pyrexia	43 (10.9)	62 (15.4)	.0747	105 (13.2)
Urinary tract infection	53 (13.5)	52 (12.9)	.8346	105 (13.2)
Decubitus ulcer	53 (13.5)	49 (12.2)	.5971	102 (12.8)
Pneumonia	47 (12.0)	50 (12.4)	.9138	97 (12.2)
Treatment-related AEs	27 (6.9)	23 (5.7)	.5599	50 (6.3)
Severe AEs	236 (60.1)	235 (58.3)	.6653	471 (59.2)
Treatment-related severe AEs	2 (0.5)	2 (0.5)	1.0000	4 (0.5)
Serious AEs	227 (57.8)	232 (57.6)	1.0000	459 (57.7)
Treatment-related serious AEs	0 (0.0)	1 (0.2)	1.0000	1 (0.1)
Medically attended AEs	347 (88.3)	356 (88.3)	1.0000	703 (88.3)
AEs occurring within 1 h after first vaccination	5 (1.3)	8 (2.0)	.5783	13 (1.6)
AEs occurring within 1 h after second vaccination	3 (0.8)	3 (0.7)	1.0000	6 (0.8)

*Abbreviation*: *AE* adverse event

^a^Patients counted only once per preferred term (MedDRA 18.1). *P* values for pairwise comparisons: Fisher’s exact test

The most frequently reported severe AEs in the IC43 100 ug and placebo groups were similar and included respiratory failure (6.9% vs 5.7%), septic shock (4.1% vs 6.5%), cardiac arrest (4.3% vs 5.7%), multiorgan failure (4.6% vs 5.5%), and sepsis (4.6% vs 4.2%). One patient of the placebo group experienced a possibly related SAE (respiratory failure); no related SAEs were reported in the IC43 100 μg group.

Overall, the proportion of patients with reported local reactions was low and comparable between the two treatment groups (< 1% of patients in both groups). In general, hematology and clinical chemistry parameters were comparable between the two treatment groups at all time points, with the exception of C-reactive protein. Compared with the IC43 100 μg group, C-reactive protein levels were slightly raised at all time points in the placebo group, but most notably at early termination visit (mean 273.4 vs 67.6 mg/L, respectively).

## Discussion

Despite the availability of new classes of antibiotics, multidrug resistance to *P. aeruginosa* has doubled over the past 30 years and improvements in the management of MDR PA pneumonia are a global challenge and unmet clinical need [17, 18]. This is the first large randomized trial to evaluate a vaccination strategy in complex medically ill ICU patients requiring MV and aiming to decrease all-cause mortality at day 28. This study proved the feasibility of vaccination and inducing specific immune responses in a complex ICU patient cohort. While the IC43 vaccine was demonstrated to be safe and immunogenic, no significant differences in all-cause mortality or survival rates vs placebo were observed.

The study was undertaken to confirm the previously observed promising effects of IC43 100 μg on all-cause mortality and to evaluate the benefits and risks of using vaccines in an ICU setting [13]. A previous phase 2 study in ICU patients requiring MV showed a significantly lower mortality rate for the IC43 group (21.5%) versus the placebo group (40%) at day 28 associated with high immunogenicity in the IC 43 group. Other trials including critically ill patients observed mortality rates of about 25%, which are lower than the mortality observed in the cohort of this phase 2 study [19]. In this context, it needs to be stated that for sample size calculation of the present IC43 trial, both the differences in mortality rate and the predicted placebo mortality rate at the time point of primary endpoint assessment (day 28) were conservatively estimated lower than observed in the phase 2 study. The missing correlation between the observed mortality benefit and confirmed *P. aeruginosa* infections in the initial phase 2 trial required a phase 3 trial. In retrospect, probably another phase 2b trial performed in a more selected population at risk (or even colonized with *P. aeruginosa*) would have been more appropriate. In addition, choosing *P. aeruginosa-*related event as a broader primary endpoint may have been an option.

In the current study, vaccination with IC43 resulted in strong and consistent humoral responses; the absence of clinical efficacy of IC43 vaccination was therefore probably not due to failure to elicit an antibody response. However, antibody responses were limited at day 7 (i.e., 16.6% SCR), before the second dose was administered. It is possible that the time course of antibody development, even at 2 weeks, is insufficient to influence clinical outcome in acute, critically ill patients. Furthermore, as stated before, the rates of patients with at least one DMC-confirmed *P. aeruginosa* infection did not differ significantly between the IC43 100 μg and the placebo group. Instead, there was even a statistically significantly higher rate of respiratory tract colonization in the vaccinated group compared to the placebo group. In the absence of any effect on *P. aeruginosa* infections, we assume this to be a chance observation, not unlikely given the number of statistical tests. The hypothesis that antibody titers elicited with the IC43 100 μg vaccine, particularly after the second vaccination, could result in a reduction of *P. aeruginosa* infections could not be confirmed. Unlike in the previous phase 2 study, in the current trial, *P. aeruginosa* was not actively screened for per protocol, and this was left to the discretion of the treating physician.

An additional difference compared to the IC43 phase 2 study was the restriction of the study population to medically ill ICU patients, in which the greatest clinical benefit could be expected. Due to the high-risk patient population studied, the mortality rate had reached roughly a fifth (17.1 vs 19.8%) of the included patients by day 14, limiting the number of potential endpoints occurring after the specific antibody titers peaked on day 28. In this context, it needs to be stated that the observed mortality rate up to the time point of primary endpoint assessment (day 28) was well within the expected mortality rate of 25 to 30%.

The high rate of systemic antibiotics already initiated before study inclusion (79% of patients received agents effective against *Pseudomonas*) potentially reduces the effect of a vaccine. The fact that most patients were receiving antibiotics prior to vaccine dosing was expected because prior antibiotic exposure is consistently identified as a risk factor for nosocomial *P. aeruginosa* pneumonia [20]. Moreover, overall, approximately 98% received concomitant antibiotics during the trial, and it will be therefore not possible to explore potential interactions between vaccination and antibiotic exposure on *P. aeruginosa* isolates.

The rates of *Pseudomonas* colonization, which occur early during an ICU stay, are also a factor mitigating against vaccine effect, particularly since they were significantly higher at baseline and throughout the study in the IC43 100 ug group. However, the observed rates of *Pseudomonas* infection and colonization were not greatly different from those closely monitored in the phase 2 study. In addition, a certain level of uncertainty remains with respect to truly distinguishing *P. aeruginosa* infection from *P. aeruginosa* airway colonization based on respiratory clinical specimens, even with the use of predefined diagnostic criteria.

A proof of efficacy for the IC43 antigen against *P. aeruginosa* has not firmly been established in humans despite several promising earlier small-scale studies [5, 15]. It is possible that the induced antibodies were not protective. Despite a number of possible targets, the high variability between *Pseudomonas* species and the complexity of its infection process and interaction with the host’s immune response has made it difficult to develop an effective vaccine difficult [9].

In a planned interim analysis conducted after 394 patients were enrolled, results demonstrated a 4.5% absolute mortality reduction in the IC43 100 ug group compared with the placebo group. Though the pre-specified nonbinding futility criterion was formally met, the DMC recommended continuation of the trial and the sponsor endorsed the decision. The attributable mortality of *P. aeruginosa* infection in mechanically ventilated patients is dependent on the severity of illness, susceptibility pattern, and appropriate antibiotic prescription, being estimated to be around 13% [21, 22]. Thus, a 10% reduction in mortality would have been very optimistic, a factor that had been taken into account for the decision to continue. In the second part of the study after the interim analysis, the trend was in fact overturned with higher overall mortality at day 28 in the active treatment group, resulting in an increase of 1.5% when compared to placebo at the final analysis. The absolute mortality rates observed in the interim analysis were well within the 95% CI limits of the mortality rates at the final analysis and were, therefore, not improbable. This is a common problem of phase 3 ICU trials where encouraging earlier analyses were reduced or abolished after larger trials in these complex study populations [23].

One of the limitations of this trial is that *P. aeruginosa* events were not screened for by protocol, but this was left to the discretion of the treating physician. It was performed according to local standards of care. Therefore, it may be possible that the number of *P. aeruginosa* events has been underestimated. The rationale for not providing antibiotic policies was chosen in order to show the effect of the vaccine on top of general practice antibiotic background therapy in this multinational trial and due to the heterogeneity of susceptibilities and mechanisms of resistance of the pathogen [24] which would require a personalized approach depending of different ICUs’ (52 sites) predominant mechanisms of resistance.

The inclusion of patients from different European countries may have introduced some variability in the current study as there is great variation in the standards of care for patients requiring long-term MV across Europe [25]. Furthermore, by including centers from different countries and additional centers than were included in the phase 2 study [13], some of which contributed only few subjects for analysis, it is possible that a change in enrollment of the patient type may have enhanced the variability of the ICU population pool. Heterogeneity of ICU populations is a recognized factor in interventional trials, and while the eligibility criteria were not altered during the current study, possible selection bias through different regional referral patterns may have led to a broader patient selection or earlier/later intervention. In line with these interpretations, the country of residence was shown to be a significant factor for survival/mortality in several regression models. Choosing a clinical and statistically significant improvement in all-cause mortality as the primary endpoint, which is of course very difficult to reach in clinical trials performed in intensive care patients, as well as the lack of an enrichment strategy in order to include a more selected ICU patient population at higher risk for *P. aeruginosa* may be considered, retrospectively, very challenging for the design of a study protocol.

Interventions to prevent nosocomial infection using a vaccine at the time point of ICU admission may come too late, and potentially, vaccination before admission would allow more time to show an effect. A large, randomized trial investigating an *S. aureus* vaccine administered weeks before elective cardiothoracic surgery failed to reach its primary endpoint [26]. It remains unclear whether vaccine candidates using other *S. aureus* antigens could be efficacious prophylactically; similarly, some subsets of patients exposed at high risk of *P. aeruginosa*, such as those requiring prolonged MV for elective surgery, could benefit from preoperative vaccination with other *P. aeruginosa* vaccine candidates. As stated above, the primary endpoint of the present study was chosen based on the results of the preceding phase 2 study. Clearly, achieving mortality reduction in a broad ICU population would have been interesting, as an impact on mortality is very difficult to demonstrate in critically ill patients. The main reason for continuing the trial after the formal, nonbinding futility criterion was met was that the observed absolute mortality difference of 4.5% represented a clinically important signal bearing the many negative previously performed ICU trials in mind.

However, the trial confirmed good immunogenicity and an acceptable safety profile. Despite evident immunogenicity between days 7 and 14, *P. aeruginosa* infection occurred prior to the development of IgG immune response. This suggests that the effectivity of pre-emptive administration before elective surgery in high-risk patients needs to be explored. The enhancement of innate and adaptive immunity remains a promising approach to vanquish emerging MDR and XDR *P. aeruginosa* [27].

## Conclusion

The present study evaluated the concept of vaccination as a strategy to reduce mortality and to prevent a potentially lethal infection with *P. aeruginosa* in ventilated non-surgical ICU patients. IC43 100 μg vaccination was both immunogenic and well tolerated; however, there was no clinical benefit of IC43 compared to placebo treatment.

## Supplementary information

**Additional file 1: eTable 1.**DMC-Confirmed P aeruginosa Respiratory Tract Infection/Colonization. **eTable 2.** SOFA Score by Visit and Treatment Group (Intent-to-Treat Population). Immunogenicity information. **eFigure 1.** Reverse Cumulative Distribution Curve to Show Proportion of Patients With an OprF/I-Specific IgG Antibody Titer Above a Specific Value at Day 28 by Treatment Group (Intent-to-Treat Population).

## Data Availability

Inquiries concerning data may be sent to the sponsor of the trial.

## References

[1] Ewans T (1999). Prevention and control of nosocomial infection in the intensive care unit.

[2] Saviteer SM, Samsa GP, Rutala WA (1988). Nosocomial infections in the elderly. Increased risk per hospital day. Am J Med.

[3] Sadikot RT, Blackwell TS, Christman JW, Prince AS (2005). Pathogen–host interactions in Pseudomonas aeruginosa pneumonia. Am J Respir Crit Care Med.

[4] Yum H-K, Park I-N, Shin B-M, Choi S-J (2014). Recurrent Pseudomonas aeruginosa infection in chronic lung diseases: relapse or reinfection?. Tuberc Respir Dis (Seoul).

[5] Morrison Allan J., Wenzel Richard P. (1984). Epidemiology of Infections Due to Pseudomonas aeruginosa. Clinical Infectious Diseases.

[6] National Nosocomial Infections Surveillance System (2004). National Nosocomial Infections Surveillance (NNIS) System Report, data summary from January 1992 through June 2004, issued October 2004. Am J Infect Control.

[7] Park DR (2005). The microbiology of ventilator-associated pneumonia. Respir Care.

[8] Porzecanski I, Bowton DL (2006). Diagnosis and treatment of ventilator-associated pneumonia. Chest.

[9] Rello J, Ramirez Estrada S, Borgatta B (2016). Pseudomonas aeruginosa ventilator-associated pneumonia management. Infect Drug Resist.

[10] Rello J, Ausina V, Ricart M (1994). Risk factors for infection by Pseudomonas aeruginosa in patients with ventilator-associated pneumonia. Intensive Care Med.

[11] Priebe GP, Goldberg JB (2014). Vaccines for Pseudomonas aeruginosa: a long and winding road. Expert Rev Vaccines.

[12] Campfield B, Chen K, Kolls JK (2014). Vaccine approaches for multidrug resistant gram negative infections. Curr Opin Immunol.

[13] Rello J, Krenn C-G, Locker G (2017). A randomized placebo-controlled phase II study of a Pseudomonas vaccine in ventilated ICU patients. Crit Care.

[14] Westritschnig K, Hochreiter R, Wallner G, Firbas C, Schwameis M, Jilma B (2014). A randomized, placebo-controlled phase I study assessing the safety and immunogenicity of a Pseudomonas aeruginosa hybrid outer membrane protein OprF/I vaccine (IC43) in healthy volunteers. Hum Vaccin Immunother.

[15] Mansouri E, Gabelsberger J, Knapp B (1999). Safety and immunogenicity of a Pseudomonas aeruginosa hybrid outer membrane protein F-I vaccine in human volunteers. Infect Immun.

[16] Suetens C, Morales I, Savey A (2007). European surveillance of ICU-acquired infections (HELICS-ICU): methods and main results. J Hosp Infect.

[17] Kollef MH, Chastre J, Fagon J-Y (2014). Global prospective epidemiologic and surveillance study of ventilator-associated pneumonia due to Pseudomonas aeruginosa*. Crit Care Med.

[18] Borgatta B, Lagunes L, Imbiscuso AT, Larrosa MN, Lujàn M, Rello J (2017). Infections in intensive care unit adult patients harboring multidrug-resistant Pseudomonas aeruginosa: implications for prevention and therapy. Eur J Clin Microbiol Infect Dis.

[19] Lemiale V, Mokart D, Resche-Rigon M (2015). Effect of noninvasive ventilation vs oxygen therapy on mortality among immunocompromised patients with acute respiratory failure. JAMA.

[20] Hoang S, Georget A, Asselineau J (2018). Risk factors for colonization and infection by Pseudomonas aeruginosa in patients hospitalized in intensive care units in France. PLoS One.

[21] Rello J, Jubert P, Vallés J, Artigas A (1996). Evaluation of outcome for intubated patients with pneumonia due to Pseudomonas aeruginosa. Clin Infect Dis.

[22] Rello J, Rué M, Jubert P, Muses G (1997). Survival in patients with nosocomial pneumonia: impact of the severity of illness and the etiologic agent. Crit Care Med.

[23] Opal SM, Dellinger RP, Vincent J-L, Masur H, Angus DC (2014). The next generation of sepsis clinical trial designs. Crit Care Med.

[24] Dimopoulos G, Akova M, Rello J, et al. Understanding resistance in Pseudomonas. Intensive Care Med. 2020. 10.1007/s00134-019-05905-6 PMC7224039 31960069

[25] Sakr Y, Moreira CL, Rhodes A (2015). The impact of hospital and ICU organizational factors on outcome in critically ill patients. Crit Care Med.

[26] Fowler VG, Allen KB, Moreira ED (2013). Effect of an investigational vaccine for preventing Staphylococcus aureus infections after cardiothoracic surgery. JAMA.

[27] Dimopoulos G, Akova M, Rello J, et al. Understanding resistance in Pseudomonas. Intensive Care Med. 2019; Epub Ahead of Print. 10.1007/s00134-019-05905-6 PMC7224039 31960069

