# Supplementary material for: Efficacy, immunogenicity, and safety of IC43 recombinant Pseudomonas aeruginosa vaccine in mechanically ventilated intensive care patients—a randomized clinical trial
Source: Crit Care. 2020 Mar 4;24:74. doi: 10.1186/s13054-020-2792-z (PMC7057595; doi:10.1186/s13054-020-2792-z)
Supplement: Supplementary file 1 — Additional file 1: eTable 1.DMC-Confirmed P aeruginosa Respiratory Tract Infection/Colonization. eTable 2. SOFA Score by Visit and Treatment Group (Intent-to-Treat Population). Immunogenicity information. eFigure 1. Reverse Cumulative Distribution Curve to Show Proportion of Patients With an OprF/I-Specific IgG Antibody Titer Above a Specific Value at Day 28 by Treatment Group (Intent-to-Treat Population). [file 13054_2020_2792_MOESM1_ESM.docx]

**Supplementary Online Content**

**Adlbrecht C, Wurm R, Depuydt P, et al. Efficacy, immunogenicity, and safety of IC43 recombinant *Pseudomonas aeruginosa* vaccine in mechanically ventilated intensive care patients- a randomized clinical trial**

**eTable 1. DMC-Confirmed *P aeruginosa* Respiratory Tract Infection**

**eTable 2. SOFA Score by Visit and Treatment Group (Intent-to-Treat Population)**

**Immunogenicity**

**eFigure 1. Reverse Cumulative Distribution Curve to Show Proportion of Patients With an OprF/I-Specific IgG Antibody Titer Above a Specific Value at Day 28 by Treatment Group (Intent-to-Treat Population)**

**Data monitoring committee (DMC) charter and protocol excerpts with detailed definition for Pseudomonas events and steps in differentiating between infection / colonization**

**eTable 1. DMC-Confirmed *P aeruginosa* Respiratory Tract Infection**

|  | **IC43 100 μg (n=393)**  **No. (%) [95% CI]** | **Placebo (n=406)**  **No. (%) [95% CI]** | **Fisher’s exact test  (*P* value)** | **Total (N=799)**  **No. (%) [95% CI]** |
| --- | --- | --- | --- | --- |
| **At least 1 respiratory tract infection** | | | | |
| Between day 7 (>) and day 14 (≤) | 9 **(2·3)** [1·2-4·3] | 7 **(1·7)** [0·8-3·5] | ·6206 | 16 **(2·0)** [1·2-3·2] |
| Between day 14 (>) and day 90 (≤) | 15 **(3·8)** [2·3-6·2] | 15 **(3·7)** [2·3-6·0] | 1·0000 | 30 **(3·8)** [2·6-5·3] |
| Between day 0 (≥) and study end (≤) | 36 **(9·2)** [6·7-12·4] | 39 **(9·6)** [7·1-12·9] | ·9036 | 75 **(9·4)** [7·6-11·6] |
|  |  |  |  |  |

Abbreviations: CI, confidence interval; DMC, data monitoring committee.

**eTable 2. SOFA Score by Visit and Treatment Group (Intent-to-Treat Population)**

|  | **IC43 100 μg (n=393)** | **Placebo (n=406)** | **Total (N=799)** |
| --- | --- | --- | --- |
| Day 9 (visit 0) |  |  |  |
| No. | 392 | 406 | 798 |
| Mean | 8·1 | 8·2 | 8·2 |
| Median | 8·0 | 8·0 | 8·0 |
| Day 7 (visit 1) |  |  |  |
| No. | 320 | 317 | 637 |
| Mean | 5·5 | 5·5 | 5·5 |
| Median | 5·0 | 5·0 | 5·0 |
| Day 14 (visit 2) |  |  |  |
| No. | 207 | 200 | 407 |
| Mean | 5·0 | 4·6 | 4·8 |
| Median | 4·0 | 4·0 | 4·0 |
| Day 28 (visit 3) |  |  |  |
| No. | 80 | 88 | 168 |
| Mean | 4·3 | 4·3 | 4·3 |
| Median | 3·0 | 4·0 | 3·5 |
| Day 56 (visit 4) |  |  |  |
| No. | 21 | 25 | 46 |
| Mean | 4·3 | 3·4 | 3·8 |
| Median | 4·0 | 3·0 | 3·0 |
| Day 180 (visit 5) |  |  |  |
| No. | 1 | 3 | 4 |
| Mean | 2·0 | 5·3 | 4·5 |
| Median | 2·0 | 3·0 | 2·5 |

Abbreviation: SOFA, Sequential Organ Failure Assessment.

**Immunogenicity**

- OprF/I-specific IgG antibody geometric mean titers in the IC43 100 μg vaccine group were 129·4 endotoxin units (EU)/mL at baseline (day 0) and 198·1 EU/mL after the first vaccination (day 7). Antibody titers increased to 1804·6 EU/mL after the second vaccination (day 14) and peaked at 2592·4 EU/mL on day 28, compared with a peak titer of 169·7 EU/mL in the placebo group (significant difference, analysis of variance model on day 28). After day 28, geometric mean titers started to decline, reaching 1143·3 EU/mL on day 56 and 425·2 EU/mL on day 180.
- For the IC43 100 μg vaccine group, the geometric mean fold increase in OprF/I titers ranged from 1·5 after the first vaccination on day 7 to a peak of 20 on day 28; at day 180 the geometric mean fold increase was 2·9 in the IC43 100 μg vaccine group.
- Reverse cumulative distributions demonstrate a clear difference between the treatment groups on day 14 to day 180, with peak OprF/I-specific antibody titers >1000 EU/mL on day 28 attained by >70% of patients immunized with IC43 100 μg (**eFigure 1**).
- At early time points (days 0 and 7), the reverse cumulative distributions showed similar results for both treatment groups.
- Results for the per-protocol population are in line with those for the intent-to-treat population.

**eFigure 1.** Reverse Cumulative Distribution Curve to Show Proportion of Patients With an OprF/I-Specific IgG Antibody Titer Above a Specific Value at Day 28 by Treatment Group (Intent-to-Treat Population)

Abbreviation: EU, endotoxin units.

**Data monitoring committee (DMC) charter and protocol excerpts with detailed definition for Pseudomonas events and steps in differentiating between infection / colonization**

1. **Protocol-provided Case Definitions:**

**Diagnostic Criteria for P. aeruginosa Infection or Colonization**

**Diagnosis of bacteremia**

Diagnosis of bloodstream infection is defined as one positive blood culture for a recognized pathogen (e.g., *P. aeruginosa*) plus presence of appropriate clinical symptoms.

**Diagnosis of urinary tract infection**

Urinary tract infection is defined as leucocyturia plus positive urine culture that is ≥10^5^ microorganisms per ml with no more than two species of microorganisms.

Leucocyturia will be defined according to **CDC/NNIS definitions for nosocomial infections, 2004:** positive dipstick for leukocyte esterase and/or nitrate. Pyuria (urine specimen with >10 WBC/mm^3^ or >3 WBC/high power field of unspun urine)

Plus the following clinical symptoms:

- Temperature >38°C or <36°C
- White blood cell count: > 12x10^9^/L or < 4.0x 10^9^/L

**Diagnosis of *P. aeruginosa* pneumonia**

Diagnosis of *P. aeruginosa* pneumonia will be made if the following diagnosis criteria for VAP according to the NNIS (National Nosocomial Infection Surveillance System) and according to criteria described by HELICS (Hospitals in Europe Link for Infection Control through Surveillance) are fulfilled:

Presence of a new or progressive lung infiltrate on chest radiography (or CT scan) plus at least two of the following:

- Temperature >38°C or <36°C
- White blood cell count: > 12x10^9^/L or < 4.0x 10^9^/L
- Purulent secretion

plus in accordance with microbiological diagnosis *P. aeruginosa* pneumonia will be graded (PN1 - PN4) as follows:

- PN1: minimally contaminated lower respiratory tract sample with quantitative culture (protected brush sample or distal protected aspirate: threshold > 10^3^ CFU/ml or BAL: threshold > 10^4^ CFU/ml
- PN2: non-protected sample (endotracheal aspirate, ETA) with quantitative culture (threshold > 10^5^ CFU/ml)
- PN3: alternative microbiological criteria (e.g., positive blood culture)
- PN4: sputum bacteriology or non-quantitative ETA

Comparison of pneumonia rates between treatment groups will consider PN1 – PN4 separately and combined.

**Diagnosis of *P. aeruginosa* tracheobronchitis**

Diagnosis of *P. aeruginosa* tracheobronchitis will be made if the following diagnosis criteria are fulfilled:

Lack of lung infiltrate on chest radiography (or CT scan) plus at least two of the following:

- Temperature >38°C or <36°C
- White blood cell count: > 12x10^9^/L or < 4.0x 10^9^/L
- Purulent secretion

plus in accordance with microbiological diagnosis *P. aeruginosa* tracheobronchitis will be graded (TB1 – TB3) as follows:

- TB1: minimally contaminated lower respiratory tract sample with quantitative culture (protected brush sample or distal protected aspirate: threshold > 10^2^ CFU/ml or BAL: threshold > 10^3^ CFU/ml
- TB2: non-protected sample (endotracheal aspirate, ETA) with quantitative culture (threshold > 10^4^ CFU/ml)
- TB3: sputum bacteriology or non-quantitative ETA

Comparison of tracheobronchitis rates between treatment groups will consider TB1 – TB3 separately and combined.

**Diagnosis of *P. aeruginosa* respiratory tract colonization**

Diagnosis of *P. aeruginosa* colonization will be made if the following criteria are fulfilled:

Positive culture from lower respiratory tract (protected brush sample, distal protected aspirate, or endotracheal aspirate) or sputum, but neither the diagnostic criteria for pneumonia nor for tracheobronchitis are fulfilled.

1. **DMC Review of the *P.aeruginosa* infections/colonizations followed general rules defined in the DMC meeting:**

- It was agreed that the DMC should focus on whether the protocol-defined criteria were met or not for their confirmation of an infection. Hence, an infection/ colonization was in general only confirmed by the DMC if all predefined criteria were met (exception: tracheobronchitis, see next bullet).
- Divergent to the predefined criteria the DMC agreed to confirmed tracheobronchitis cases if “Lack of new or progressive lung infiltrate on chest radiography or CT scan” was answered “no” or “not available” but criterion “purulent secretion” was answered “yes” (and all other predefined criteria were met), for the following considerations: Presence of purulent secretion was considered clearly indicative of an infection even in the absence of an X-ray or CT scan; and investigators may have been tempted to answer the question “lack of new or progressive lung infiltrate... “ with “no” when there was no such infiltrate.
- If for a patient a *P.ae.* infection and *P.ae.* colonization have been reported with same start dates and diagnostic criteria for all infection types were met, the more severe event (pneumonia > tracheobronchitis > respiratory tract colonization) was confirmed, whereas the less severe infection / colonization was not confirmed. If, however, different infection types were reported with different start dates in the same patient, both infection types could be confirmed by the DMC if respective criteria were met.
- If a tracheobronchitis could not be confirmed by the DMC because not all pre-defined diagnostic criteria were met, the tracheobronchitis was “downgraded” to a respiratory tract colonization (in line with the diagnostic criteria in the protocol). For such cases, the start date as assessed by the investigator for the tracheobronchitis will be used as the start date for the respiratory tract colonization.
- If a pneumonia (PN) could not be confirmed by the DMC, the PN was “downgraded” to either tracheobronchitis or respiratory tract colonization, depending on which criteria were met. The start date as assessed by the investigator for the PN will be used as the start date for the tracheobronchitis/ respiratory tract colonization.
